# Involuntary movements of pelvic stump

**DOI:** 10.1002/ccr3.909

**Published:** 2017-03-29

**Authors:** Carmen García‐Cabo Fernández, Laura Martínez‐Rodríguez, Pedro Oliva‐Nacarino, Germán Morís de la Tassa

**Affiliations:** ^1^ Neurology DepartmentHospital Universitario Central de AsturiasOviedoAsturiasSpain

**Keywords:** Convulsive movements, involuntary movements, limb amputation, phantom phenomena, stump

## Abstract

Although involuntary movements of stumps are less frequent than phantom sensation or other neurological sequelae of limb amputation, they represent a phenomenon that has been known for many years. The pathophysiology remains unknown, but it seems to be related to damage to the peripheral nervous system. Treatment is not standardized, but antimyoclonic drugs seem to be useful.

## Introduction

Although the most frequently described neurological sequelae of limb amputation are phantom sensations, the occurrence of involuntary movements of stumps is a phenomenon that has been known for many years, but they have not often been described 1, 2.

Positive involuntary movements in amputation stumps are choreiform or myoclonic movements in the remaining limb after amputation. The pathophysiology has not been completely elucidated, but the condition appears to represent a variant in the spectrum of movement disorders induced by an injury to the peripheral nervous system 3.

## Case Report

We describe a 66‐year‐old Spanish patient whose past medical history included malignant fibrous histiocytoma of pelvis diagnosed 4 years ago. He underwent several operations for the resection of the tumor and left hemipelvectomy were performed due to a tumor recurrence.

A few days after surgery, he developed involuntary movements of the pelvic stump, but pain or phantom sensations were denied by the patient. There were no other neurological symptoms and no relevant neurological past or family history, he was on no medications.

Clinical examination showed rhythmic, short, spontaneous, and involuntary jerks of the stump (see Video S1). Abnormal neurological movements were confined to the left hemipelvis.

Computer tomography of the brain and magnetic resonance imaging of the spinal cord were normal. A routine electroencephalography (EEG) and somatosensory evoked potentials (SSEP) did not show any abnormalities despite the fact that the involuntary movements were occurring at the time of the test. Electromyographic (EMG) recording of muscle activity was not carried out because the involuntary movements resolved after 24 h to low doses of levetiracetam (500 mg bd).

## Discussion

Whereas most reported cases of motor impairment after limb amputation are related to functional impotence, there are cases and series of cases that reported abnormal movements in amputation stumps 4, 5. These abnormal movements frequently occur in the postsurgical period and are settled over days or even months 5. In most cases jerking of the stump was preceded by the development of pain in the amputation limb, but there are case reports of involuntary movements of stumps with no pain reported. Although this syndrome etiology is not clear, some authors support that stump involuntary movements may represent a form of peripheral myoclonus caused by injury of peripheral nerve damage, and it is postulated that they may be caused by the activation of spinal interneuronal circuits 3.

Peripheral movement disorders such as myoclonus, athetosis, and dystonia have been infrequently described and poorly understood. The pathophysiology has not been completely elucidated. Some authors proposed that a neuroanatomical network linking the somatic and sympathetic nervous system with participation of substance P directly initiates the abnormal depolarization of anterior horn cells.

Myoclonus is caused by the brief activation of one muscle, leading to a jerk of the affected body part. This activation can arise from the cortex, subcortical structures, spinal cord or nerves roots, and plexi. In cortical myoclonus jerks will be preceded by a cortical discharge, which can be frequently recorded on EEG, although sometimes this test has limited sensitivity, and very high amplitude cortical somatosensory evoked potentials are usually recorded.

We assumed that the involuntary movements were a form of peripheral myoclonus on the account that EEG, SSEP and the imaging of the spinal cord were normal. Unfortunately, this assumption could not be backed up by EMG data.

As the pathophysiology is unknown, the treatment is not standardized, although drugs to treat myoclonus seem to be useful. There are also cases in which pramipexole treatment led to a marked decrease in the episodes 6. Other cases treated with clobazam have also had good clinical results4. In the patient we described, levetiracetam led to a complete resolution of the symptoms.

## Authorship

CGCF: wrote the manuscript. LMR and PON: participated in the diagnosis and treatment of the patient described. GMT: reviewed the manuscript and advised about scientific content.

## Conflict of Interest

All authors have not any actual or potential conflict of interest.

## Supporting information


**Video S1.** Involuntary movements of the left pelvic stump.
